# Decoding Diabetes: Hub Genes as Pivotal Players in Cardiomyopathy and Kidney Disease

**DOI:** 10.2174/0118715303393363251028113435

**Published:** 2026-01-15

**Authors:** Jiarong Liu, Wen Chen, Yun Zou, Jixiong Xu

**Affiliations:** 1 Department of Endocrine and Metabolism, The First Affiliated Hospital, Jiangxi Medical College, Nanchang University, Nanchang, Jiangxi, People's Republic of China;; 2 Jiangxi Clinical Research Center for Endocrine and Metabolic Disease, Nanchang, Jiangxi, People's Republic of China;; 3 Jiangxi Branch of National Clinical Research Center for Metabolic Disease, Nanchang, Jiangxi, People's Republic of China

**Keywords:** Diabetic kidney disease, diabetic cardiomyopathy, differentially expressed genes, bioinformatics analysis, *in vivo/in vitro* experiments, hyperglycemia

## Abstract

**Introduction:**

Diabetes mellitus (DM) induces systemic vascular dysfunction and leads to life-threatening complications, including diabetic cardiomyopathy (DCM) and diabetic kidney disease (DKD), whose shared mechanisms remain elusive. In this study, we performed an in- depth bioinformatics analysis to identify shared therapeutic targets and key molecular players in DKD and DCM.

**Methods:**

Integrated computational and experimental approaches were employed. Bioinformatics analysis of GEO datasets (GSE30122 and GSE197850) identified differentially expressed genes (DEGs). Hub genes were extracted *via* protein-protein interaction networks and functional enrichment. *In vitro* validation was performed using AGE-stimulated cardiomyocytes and podocytes analyzed by qPCR, complemented by *in vivo* studies in rat models. Additionally, protein-chemical interactions and drug repurposing analyses were performed.

**Results:**

We identified 48 common DEGs (*p*-adj< 0.05 and |log_2_FC| > 1.0) and prioritized 7 hub genes (CD200, CRHBP, DHRS3, EMCN, HPGD, PDGFRB, and SULF1), which were validated as dysregulated in *in vitro* and *in vivo* models. Computational screening revealed 10 promising therapeutic candidates (*p*-adj < 0.05) targeting core pathogenic networks.

**Discussion:**

Our study is one of the first to simultaneously investigate the molecular underpinnings of DKD and DCM by integrating bioinformatics data with experimental validation. Meanwhile, the relatively small sample sizes may limit the statistical power and generalizability of the identified DEGs.

**Conclusion:**

This study uncovers novel shared mechanisms between DCM and DKD, providing a framework for dual-organ protective therapies to advance the management of diabetic complications.

## 1 INTRODUCTION

With societal progress, changes in lifestyles, and the continuous rise in diabetes mellitus (DM) cases, DM has become a significant global public health issue, placing considerable strain on both physical and mental health as well as socio-economic systems. As of 2021, there were 529 million diabetes patients worldwide, with an age-standardized prevalence rate of 6.1%. It is estimated that by 2050, the number of diabetes patients will exceed 1.31 billion [1-3]. Treating diabetes is challenging not only due to difficulties in controlling hyperglycemia but also because of the multiple complications that hyperglycemia induces in various organs, blood vessels, nerves, and the immune system [4, 5].

Diabetic cardiomyopathy (DCM), the most common cardiovascular complication of diabetes, was first reported by S. Rubler in 1972 [6] and has since become one of the leading causes of death among DM patients [7, 8]. DCM is typically characterized by myocardial fibrosis, left ventricular hypertrophy, impaired remodeling, and diastolic dysfunction in its early stages, which later progress to systolic dysfunction and reduced ejection fraction, ultimately resulting in heart failure [9, 10]. Key mechanisms in the pathophysiology of DCM include oxidative stress, glucotoxicity, lipotoxicity, deposition of advanced glycation end products (AGEs), inflammation, cardiac autonomic neuropathy, and microvascular dysfunction [11, 12]. Yet, in the past 50 years, little progress has been made in the prevention, diagnosis, and treatment of DCM [13, 14].

The kidney is arguably the most critical target of microvascular damage in DM. Diabetic kidney disease (DKD) is a severe microvascular complication and the leading cause of end-stage renal disease (ESRD) [15, 16]. High blood glucose, oxidative stress, persistent inflammation, accumulation of AGEs, lipid metabolism disorders, and abnormal cellular processes affect multiple kidney cell types, leading to various forms of cell death and consequent structural and functional damage. Clinically, patients with advanced DKD often present with myocardial lesions. Significant interactions among DM, heart failure, and renal dysfunction create vicious cycles, and breaking these cycles represents a critical therapeutic goal [17, 18]. Moreover, pharmaceutical interventions beneficial for the general population may be ineffective or even harmful in this high-risk group. Therefore, clinicians managing DM patients with DCM and DKD must be aware of these complexities and adopt individualized treatment strategies [19].

In recent years, the use of hypoglycemic and other novel drugs has gradually revealed their benefits for both the heart and the kidneys [20, 21]. Nevertheless, identifying precise therapeutic targets and developing targeted drugs for the effective treatment of DKD with concurrent DCM remains challenging. To simultaneously address multiple diabetic complications, a better understanding of the underlying molecular pathways and potential therapeutic targets is essential.

In this study, we performed an in-depth bioinformatics analysis to identify shared therapeutic targets and key molecular actors in DKD and DCM. Hub genes among the differentially expressed genes (DEGs) were identified using Cytoscape and subsequently validated by qPCR in mouse podocyte (MPC5) and rat cardiomyoblast (H9C2) cell lines. Additionally, we established *in vivo* rat models of diabetic nephropathy and cardiomyopathy. Following tissue harvesting and histological staining, qPCR experiments were conducted at the tissue level to verify hub gene expression. Based on the identified hub genes, potential drug candidates were screened using the Drug Signatures Database (DSigDB). Elucidating common therapeutic targets is vital for developing new therapeutic strategies and for providing valuable insights into designing targeted therapies to address the interconnected nature of DKD and DCM. The detailed research methodology for this study is illustrated in  Fig. (**1**).

## 2 MATERIALS AND METHODS

### 2.1 Dataset Download

We searched the Gene Expression Omnibus (GEO) database (http://www.ncbi.nlm.nih.gov/geo/) using the keywords “diabetes kidney disease” (or “diabetic nephropathy”) and “diabetic cardiomyopathy” and obtained two mRNA expression microarray datasets related to DKD and DCM (GSE30122 and GSE197850) [22, 23]. The GSE30122 dataset includes nine DKD samples and 26 healthy controls, while GSE197850 comprises 4 DCM samples and 4 healthy controls. GSE30122 is based on the GPL571 Affymetrix Human Genome U133A 2.0 Array platform, and GSE197850 is based on the GPL11154 Illumina HiSeq 2000 (Homo sapiens) platform. The raw count matrices were log_2_-transformed using Excel to meet the NetworkAnalyst file upload requirements.

### 2.2 Identification of DEGs through Data Pre-processing and Screening

To compare the two datasets, we analyzed each separately using the R package “limma” (Version 4.3.1). Probe sets lacking corresponding gene symbols were excluded, and for genes represented by multiple probe sets, the expression values were averaged. DEGs were identified using a filtering criterion of |log_2_FC| > 1 and an adjusted *p*-value of < 0.05. DEGs were visualized using heatmaps and volcano plots generated with the R packages “ComplexHeatmap” and “ggplot2”, respectively. A Venn diagram (http://www.ehbio.com/test/venn/#/) was used to depict the overlapping DEGs between DKD and DCM. The intersections of upregulated and downregulated DEGs in the two datasets were considered candidate DEGs for further analysis.

### 2.3 Enrichment Analysis

Gene Ontology (GO) functional enrichment analysis [24, 25] and Kyoto Encyclopedia of Genes and Genomes (KEGG) pathway enrichment analysis [26, 27] were performed using the “clusterProfiler” package in R (Version 4.3.1) to assess the biological functions of the 48 overlapping DEGs. An adjusted *p*-value < 0.05 was considered statistically significant, and the top 10 results from each analysis were extracted for visualization [26-28].

### 2.4 Protein-Protein Interaction (PPI) Network Analysis and Identification of Hub Genes

A PPI network was constructed using the STRING database (http://string-db.org) to examine interactions among overlapping DEGs [29, 30]. Interactions with a score above 0.4 were deemed significant, and the network was visualized with Cytoscape (Version 3.10.1). Key modules were identified using the Cytoscape plugin MCODE (Minimal Common Oncology Data Elements) [31, 32]. Four different algorithms were applied to the PPI network to identify hub genes with the highest connectivity.

### 2.5 Verification of Hub Gene Expression in *In vitro* Cell Experiments

#### 2.5.1 Preparation and Characterization of Gly-AGEs

To prepare Gly-AGEs, D-(+)-glyceraldehyde (Sigma-Aldrich, USA) was dissolved in 0.2 mol/L PBS to a final concentration of 0.1 mol/L. After filtration through a 0.22-µm filter, the solution was maintained at 37°C in a humidified atmosphere containing 5% CO_2_ for 7 days, while non-glycosylated BSA served as a blank control. Subsequently, dialysis was performed with 0.2 mol/L PBS for 2 days to remove unbound D-(+)-glyceraldehyde. The dialyzed AGEs were filtered again through a 0.22 µm membrane to remove bacteria, and the AGE concentration was determined and stored at 4°C. The whole preparation process needed to be protected from light.

#### 2.5.2 Cell Culture

The MPC5 and H9C2 cell lines were purchased from Shanghai Fuheng Biotechnology Co., Ltd (Shanghai, China) and cultured in DMEM (Gibco, USA) supplemented with 10% fetal bovine serum (FBS).

#### 2.5.3 Cell Viability Assay

Cell viability was assessed using the Cell Counting Kit-8 (CCK-8, Glpbio, USA) [33, 34]. MPC5 and H9C2 cells were seeded in 96-well plates and treated with various concentrations of AGEs for 1, 2, 3, and 4 days, respectively. Then, 100 μL of complete medium containing 10 μL of CCK-8 reagent was added to each well. After a 2-hour incubation at 37°C, absorbance was measured using a microplate spectrophotometer (Varioskan LUX, Thermo Fisher). Cell viability assays were conducted with six replicates per concentration group in three independent experiments. Each experiment used freshly prepared AGEs solutions and newly passaged cells (passages 3-8). Data represent mean ± SD of three biological replicates.

#### 2.5.4 qPCR

Total RNA was extracted using TRIzol (Invitrogen, Thermo Fisher Scientific). After confirming mRNA purity, cDNA was synthesized *via* reverse transcription. qPCR reactions were performed on a BIO-RAD CFX96 detection system using TransStart Top Green qPCR SuperMix (TransGene Biotech) [35, 36]. Primer sequences are provided in Table **S1**. Relative mRNA levels were calculated using the 2^(-ΔΔCT) method with β-actin as the normalization control. The mRNA expression of the hub genes was validated in AGEs-treated MPC5 and H9C2 cells. Total RNA extraction and qPCR validation were performed with three independent biological replicates per experimental group. Each biological replicate consisted of triplicate technical replicates for all target genes (CD200, CRHBP, DHRS3, EMCN, HPGD, PDGFRB, SULF1) and the reference gene (β-actin). Inter-assay variability was controlled by running all samples of the same gene target on the same PCR plate. Group comparisons were conducted using a t-test; *p* < 0.05 was considered statistically significant.

### 2.6 Verification of Hub Gene Expression in *In vivo* Animal Experiments

#### 2.6.1 Animal Modeling and Grouping

Thirty healthy male SD rats (7-8 weeks old, 180-200 g, SPF grade) were purchased from Jiangsu Jicui Yaokang Biotechnology Co., Ltd. (Animal License Number: SCXK (Su) 2023-0009) and housed in the constant temperature animal laboratory of The First Affiliated Hospital of Nanchang University. Randomization was performed by an independent researcher using computer-generated random numbers. Rats were assigned unique IDs and allocated to control (n=10), DKD (n=10), or DCM (n=10) groups *via* stratified randomization based on baseline body weight. All rats were maintained in an SPF environment at 20-25°C and 56% humidity, with free access to water and a 12-hour light/dark cycle. After one week of acclimatization, rats in the DKD and DCM groups were fasted and, on the second day, received an intraperitoneal injection of streptozotocin (Sigma-Aldrich, USA) at 60 mg/kg. Seventy-two hours after STZ injection, tail vein blood glucose was measured; three consecutive readings above 16.7 mmol/L indicated successful establishment of the diabetes model. Control rats received an equivalent volume of sodium citrate buffer. All experimental procedures involving animals were conducted in strict compliance with the ARRIVE guidelines and the National Institutes of Health (NIH) Guide for the Care and Use of Laboratory Animals. This study was approved by the Institutional Animal Ethics Committee of The First Affiliated Hospital of Nanchang University (Approval No.: CDYFY-IACUC-202401QR003). Every effort was made to minimize animal suffering, reduce sample sizes, and employ alternative *in vitro* approaches where possible, adhering to the 3Rs principle.

Gene expression data analyses were based on publicly available datasets (GSE30122 and GSE197850) from the GEO database, in accordance with anonymized data use standards. Commercially available cell lines (MPC5 and H9C2) were used in the *in vitro* experiments, and no additional human samples were collected or involved in this study.

#### 2.6.2 Functional Testing of Rat Kidneys and Hearts

Weekly, changes in diet, water intake, urine output, body weight, and blood glucose were monitored and recorded. Eight weeks post-modeling, urine from DKD group rats was collected to measure urinary creatinine and microalbumin, from which the urinary albumin/creatinine ratio (UACR) was calculated. A UACR > 30 mg/g indicated kidney damage, confirming successful DKD modeling. Similarly, eight weeks after modeling, rats in the DCM group were anesthetized *via* intraperitoneal injection of 4% chloral hydrate, and cardiac function was assessed using a Philips 7500 digital echocardiogram (probe frequency: 10-12 MHz). Significant changes in cardiac function confirmed the successful establishment of the DCM model.

#### 2.6.3 Animal Sacrifice, Sampling, Staining, and qPCR Experiments

After the experimental period, rats were anesthetized using an intraperitoneal injection of pentobarbital sodium to minimize suffering. Blood was collected from the heart, allowed to clot, centrifuged, and the supernatant was stored at -80°C. Following euthanasia *via* cervical dislocation, the hearts and kidneys were quickly harvested and fixed in 4% paraformaldehyde. The fixed tissues were processed into paraffin sections and stained with HE and Masson's trichrome for histopathological evaluation under a light microscope. Subsequently, total RNA was extracted from the remaining kidney and heart tissues using TRIzol, and qPCR was performed to verify hub gene expression. All histological analyses and qPCR experiments were conducted by investigators blinded to group assignments. Tissue sections were coded by a third party, and data analysis was performed without knowledge of experimental groups until statistical validation was complete. Primer sequences are listed in Table **S1**.

### 2.7 Immune Infiltration Analysis

To quantify the proportions of 22 immune cell types, the CIBERSORT package was used with 1000 iterations on the LM22 gene signature [37, 38]. Samples with an adjusted *p*- value < 0.05 were retained, and the resulting immune cell infiltration matrix was calculated using the MOABS algorithm. Boxplots were generated to compare immune cell compositions between groups. Additionally, the correlation between the seven hub genes and the 22 immune cell types was assessed using the R package “corrplot”, and a correlation heatmap was generated with the “pheatmap” package (https://CRAN.R-project.org/package=pheatmap).

### 2.8 Protein-chemical Interactions and Gene-drug Interactions

Using NetworkAnalyst, we analyzed interactions between the seven hub genes and chemicals based on the Comparative Toxicogenomics Database (CTD). Furthermore, drug-hub gene interactions were identified using the Enrichr platform and the Drug Signatures Database (DSigDB) [39].

### 2.9 Statistical Analysis

A statistical analysis of gene expression differences between the two groups was performed using the R package ggpubr. We used R version 4.3.1 to perform all gene expression and statistical analyses. To compare two groups, a two-sample independent t-test was performed for normally distributed data, whereas the Wilcoxon test was applied for non-normally distributed data. Statistical significance was determined by a *p*-value below 0.05. All statistical analyses were completed through SPSS software (Version 26.0).

## 3 RESULTS

### 3.1 Identification of DEGs in DKD and DCM

We downloaded the DKD and DCM datasets (GSE30122 and GSE197850) from the GEO database. The GSE30122 dataset yielded 508 DEGs (113 upregulated and 395 downregulated) using an adjusted *p* < 0.05 and |log_2_FC| > 1.0 (Fig. **2A**). The GSE197850 dataset identified a total of 1296 DEGs, including 510 upregulated and 786 downregulated genes (Fig. **2B**). A Venn diagram analysis revealed 48 overlapping DEGs (Fig. **2C**), and heatmaps of these overlapping DEGs are shown in Figs. (**2D** and **2E**). Table **S2** provides basic information about these common DEGs.

### 3.2 Enrichment Analysis

GO and KEGG pathway enrichment analyses were performed on the 48 common DEGs. Based on an adjusted *p* < 0.05, the top six significantly enriched GO terms were selected. In the biological process category, the DEGs were mainly associated with vasculogenesis, muscle tissue development, coronary vasculature morphogenesis, and positive regulation of transforming growth factor beta. In terms of molecular function, the DEGs were primarily enriched in platelet-derived growth factor receptor binding, collagen binding, and glycosaminoglycan binding (Fig. **3A** and Table **S3**).

### 3.3 PPI Network Construction and Hub Gene Identification

To assess the interactions among the overlapping DEGs, a PPI network was constructed using the STRING database and visualized in Cytoscape (Fig. **3B**).

Using the betweenness ranking method, the top ten hub genes were identified as: platelet-derived growth factor receptor beta (PDGFRB), 15-hydroxyprostaglandin dehydrogenase (HPGD), nuclear receptor subfamily 3 group C member 2 (NR3C2), dehydrogenase/reductase 3 (DHRS3), sulfatase 1 (SULF1), endomucin (EMCN), corticotropin-releasing hormone binding protein (CRHBP), CD200 molecule (CD200), integrin subunit alpha 8 (ITGA8), and LIM domain binding 2 (LDB2) (Fig. **4A**).

Using the EPC ranking method, the top ten genes identified were PDGFRB, HPGD, EMCN, SULF1, DHRS3, ITGA8, NR3C2, CD200, cyclin-dependent kinase inhibitor 1C (CDKN1C), and CRHBP (Fig **4B**).

MCC ranking identified PDGFRB, EMCN, ITGA8, HPGD, CDKN1C, DHRS3, SULF1, CRHBP, Procollagen C-endopeptidase enhancer 2 (PCOLCE2), and CD200 (Fig. **4C**).

MNC ranking yielded PDGFRB, HPGD, ITGA8, DHRS3, SULF1, EMCN, CDKN1C, CD200, CRHBP, and transforming growth factor beta receptor 3 (TGFBR3) (Fig. **4D**).

The overlap among these methods identified seven hub genes: CD200, CRHBP, DHRS3, EMCN, HPGD, PDGFRB, and SULF1 (Fig. **4E**).

### 3.4 Hub Gene Expression and Validation *In vitro* and *In vivo*

Based on GEO dataset analyses (GSE30122 for DKD and GSE197850 for DCM), the DKD and DCM samples exhibited significant downregulation of all seven hub genes (CD200, CRHBP, DHRS3, EMCN, HPGD, PDGFRB, and SULF1) compared with controls (Table **1**).

*In vitro*, a CCK8 assay demonstrated that treatment with 200 μg/mL AGEs for 48 hours significantly reduced the viability of MPC5 (Fig. **5A**) and H9C2 cells (Fig. **5B**). Cell models for DKD and DCM were then established, and qPCR confirmed that the hub genes were markedly downregulated in both models (Figs. **6A** and **6B**).

*In vivo*, rat models of DKD and DCM were constructed. Histological analysis (HE and Masson staining) revealed that DKD rat kidneys displayed thickened glomerular basement membranes, tubular atrophy, and interstitial fibrosis (Figs. **7A** and **7B**), whereas DCM rat hearts exhibited enlarged, disorganized myocardial cells with extensive inflammatory cell infiltration (Figs. **7C** and **7D**). qPCR analysis of kidney and heart tissues further confirmed the significant downregulation of the seven hub genes in both complications (Figs. **8A** and **8B**). These experimental findings are consistent with the GEO dataset results.

### 3.5 Functional Enrichment Analysis of Proteins Interacting with Hub Genes

To gain insight into the shared mechanisms of DKD and DCM, GO and KEGG analyses were performed on proteins interacting with the hub genes. GO analysis revealed associations with extracellular matrix (ECM) organization, proteolytic metabolism, and cellular responses to growth factor beta. KEGG analysis indicated involvement in focal adhesion, the PI3K-Akt signaling pathway, ECM-receptor interaction, and the AGE-RAGE signaling pathway in diabetic complications. Collagen-binding and glycosaminoglycan-binding activities are associated with extracellular matrix (ECM) remodeling and fibrosis, which are central pathological processes in DCM and DKD. Positive regulation of transforming growth factor beta (TGF-β) is a critical pathway driving fibrosis and inflammation in both organs. Figs. (**S1A-G**) displays the top 10 enriched pathways.

### 3.6 Differential Expression Analysis of Immune Cell Infiltration

To explore the common immune microenvironment in DKD and DCM, we evaluated the association between hub gene expression and immune cell components using the CIBERSORT algorithm. Principal component analysis (PCA) demonstrated apparent differences in immune cell infiltration between DKD/DCM patients and their respective controls (Figs. **9A** and **9B**). Surprisingly, by separately constructing immune profiles for DKD and DCM patients and analyzing the proportions of invasive immune subtypes, we discovered significant differences in immune cell infiltration patterns between DKD and DCM (Figs. **9C** and **9D**). Notably, separate immune profiling revealed that, in the DKD group, memory B cells, naive CD4 T cells, gamma delta T cells, and resting mast cells were elevated compared with controls, while naive B cells and neutrophils were reduced (Fig. **10A**). In the DCM group, CD8^+^ T cells, M2 macrophages, and resting dendritic cells were increased, whereas naive B cells, naive CD4 T cells, and resting NK cells were decreased (Fig. **10B**). Correlation analysis showed that in DKD, the downregulated hub genes were positively correlated with naive B cells and resting memory CD4 T cells, but negatively correlated with naive T cells, M2 macrophages, and resting mast cells (Fig. **10C**). In DCM, these genes were positively correlated with naive B cells, follicular helper T cells, and resting NK cells, and negatively correlated with plasma cells, regulatory T cells (Tregs), and M2 macrophages (Fig. **10D**). Therefore, we speculate that M2 macrophages may become drivers of organ damage by promoting fibrosis in a long-term, chronic high-glucose environment, leading to heart and kidney injury. These correlations suggest that the hub genes are significantly linked to immune cell infiltration patterns.

### 3.7 Interactions between Proteins and Chemicals

Using NetworkAnalyst and the CTD database, we analyzed protein-chemical interactions (Fig. **11A**). Various chemicals were found to interact with hub genes, including valproic acid (VPA), silicon dioxide, and copper. Table **2** summarizes the top 10 chemicals that interact with 7 hub genes.

### 3.8 Gene-drug Interactions

To further explore potential drugs for the treatment of both DKD and DCM, the DSigDB database was used to screen for drugs that interact with the hub genes. The top 10 drug molecules were selected based on adjusted *P*-values, including pharmacologically active compounds, such as curcumin, embelin, and benzbromarone, as well as other chemical entities, like pentabromophenol, purpurin, pentachlorophenol, and computational identifiers from ChemBL (*e.g.*, benzbromarone BOSS) (Fig. **11B**). Table **3** summarizes the gene-drug interaction enrichment results for these candidates. The positive associations between these drug candidates and the hub genes suggest their potential utility in treating both conditions.

## 4 DISCUSSION

In this study, we conducted an integrated bioinformatics analysis, coupled with *in vitro* and *in vivo* validations, to identify common therapeutic targets underlying diabetic kidney disease (DKD) and diabetic cardiomyopathy (DCM). By analyzing two independent GEO datasets (GSE30122 for DKD and GSE197850 for DCM), we identified 48 overlapping differentially expressed genes (DEGs) and further narrowed them to seven key hub genes, namely CD200, CRHBP, DHRS3, EMCN, HPGD, PDGFRB, and SULF1, using several network topology algorithms. Subsequent validation in AGE-treated MPC5 and H9C2 cell models, as well as in streptozotocin-induced rat models, confirmed that these hub genes were significantly downregulated in both DKD and DCM. Additionally, pathway enrichment analyses, immune cell infiltration studies, and protein-chemical/gene-drug interaction networks provided further insight into the shared molecular mechanisms and potential intervention strategies for these diabetic complications.

Our study is one of the first to simultaneously investigate the molecular underpinnings of DKD and DCM by integrating bioinformatics data with experimental validation. Previous studies have largely focused on these complications in isolation [40]; however, our approach emphasizes their interconnected nature. The identification and experimental confirmation of seven consistently downregulated hub genes across both models underscore a common pathological link between DKD and DCM. This dual validation not only reinforces the reliability of the bioinformatics predictions but also suggests that these hub genes might serve as potential biomarkers or therapeutic targets for managing multiple diabetes-related complications simultaneously.

Extensive research has documented the roles of oxidative stress, chronic inflammation, and extracellular matrix (ECM) remodeling in the pathogenesis of both DKD and DCM [41-43]. However, most earlier investigations have addressed these processes in the context of a single diabetic complication. Our study expands upon this foundation by revealing that key molecular pathways, such as the AGE-RAGE and PI3K-Akt signaling pathways, are shared between DKD and DCM. The downregulation of the identified hub genes in both datasets is consistent with the notion that impaired ECM remodeling and vascular integrity, along with aberrant inflammatory responses, are central to the development and progression of these conditions.

Each of the identified hub genes appears to contribute to the pathophysiology of DKD and DCM through distinct yet interrelated mechanisms. PDGFRB (Platelet-Derived Growth Factor Receptor Beta) plays a crucial role in vascular integrity and pericyte function [44]. Studies have shown that PDGFRB can regulate coronary vessel development and revascularization during heart regeneration [45, 46]. Its downregulation may lead to compromised microvascular stability [47], thereby exacerbating capillary rarefaction and tissue ischemia in both the kidney and the heart. This vascular dysfunction can further promote fibrosis and progressive organ damage [48, 49]. As a key enzyme in prostaglandin metabolism, HPGD (15-Hydroxyprostaglandin Dehydrogenase) modulates inflammation and fibrosis [50]. Studies have reported that HPGD overexpression regulates proliferative activity, apoptosis levels, adhesion, and angiogenic capability [51]. Reduced expression of HPGD might result in the accumulation of pro-inflammatory prostaglandins, which, in turn, could trigger an amplified inflammatory response and promote excessive ECM deposition, contributing to both renal and myocardial fibrosis [52, 53]. CD200 (Cluster of Differentiation 200) is known for its immunomodulatory function, particularly in suppressing macrophage activation [54, 55]. Studies reported that CD200-CD200R signaling can attenuate high-glucose-induced inflammation and phagocytosis [56]. The CD200 checkpoint, a key limiting factor in monopoiesis, monocyte-macrophage activation, and recruitment in atherosclerosis, can serve as a novel therapeutic target for cardiovascular diseases [57]. The observed downregulation of CD200 may remove this inhibitory effect, leading to enhanced inflammatory cell infiltration and sustained inflammatory responses in diabetic tissues, thereby worsening tissue injury. CRHBP (Corticotropin-Releasing Hormone Binding Protein) modulates the activity of stress hormones [58]. Altered expression of CRHBP may disrupt the balance of the hypothalamic-pituitary-adrenal axis, thereby exacerbating metabolic disturbances and promoting stress-induced tissue damage [59]. This imbalance could be particularly deleterious in the context of chronic hyperglycemia. Moreover, some studies have shown that CRHBP is related to the cognitive ability of patients with type 2 diabetes [60]. Both EMCN (Endomucin) and SULF1 (Sulfatase 1) are involved in maintaining endothelial function and regulating ECM organization. EMCN contributes to endothelial cell adhesion and vascular barrier function [61], while SULF1 modulates the sulfation patterns of glycosaminoglycans, affecting cell signaling and ECM composition [62]. Their downregulation may impair vascular repair mechanisms and promote structural remodeling [63, 64], which are hallmarks of both DKD and DCM. There are studies demonstrating that the endothelial marker EMCN plays a critical role in supporting normal glomerular filtration barrier structure and function by maintaining glomerular endothelial tight junctions and homeostasis, and podocyte function through endothelial-podocyte crosstalk [65]. DHRS3 (Dehydrogenase/Reductase 3) is implicated in retinoid metabolism, a pathway essential for cell differentiation and tissue repair [66]. A decrease in DHRS3 expression could disrupt these processes, thereby facilitating fibrosis development and impairing the regenerative capacity of affected organs.

Immune cell infiltration analysis revealed distinct alterations in the immune microenvironment of DKD and DCM tissues. In DKD, increased infiltration of certain immune cells, coupled with reductions in others, suggests a shift toward a pro-inflammatory milieu, whereas DCM exhibits a distinct immune signature characterized by elevated CD8^+^ T cells. The significant correlations between hub gene expression and specific immune cell subsets imply that these genes may influence local immune responses, contributing to inflammation and subsequent fibrotic changes.

The functional enrichment analyses underscored the involvement of several critical signaling pathways, including the AGE-RAGE, PI3K-Akt, and ECM-receptor interaction pathways. These pathways have been previously implicated in the pathogenesis of diabetic complications, providing a mechanistic rationale for our findings. For instance, the AGE-RAGE pathway triggers inflammatory cascades and oxidative stress, whereas the PI3K-Akt pathway is essential for cell survival and proliferation. The modulation of these pathways through the identified hub genes opens new therapeutic avenues. Our drug prediction analysis, based on the DSigDB database, identified candidate drugs (such as curcumin, embelin, and pentabromophenol) that may interact with these hub genes.

For example, curcumin, the major component of the rhizome of *Curcuma longa,* has various pharmacological actions, including anti-inflammatory, immunomodulatory, antioxidant, and lipid-modifying properties. In general, the biological effects of curcumin are broadly thought to be achieved by inhibition of NF-κB [14, 67]. In addition, evidence from evidence-based medicine shows that curcumin has anti-diabetic activity through its ability to inhibit oxidative stress and inflammatory processes [68, 69]. Embelin, a benzoquinone, is one such agent isolated from *Embelia ribes*, which has shown excellent biological activities toward several chronic ailments, including diabetes, by upregulating several antioxidant enzymes, inhibiting anti-apoptotic genes, modulating transcription factors, blocking inflammatory biomarkers, monitoring cell cycle synchronizing genes, *etc.* [70-72]. Additionally, studies have reported that purpurin ameliorated neuropathic allodynia and hyperalgesia by modulating neuronal mitochondrial bioenergetics and redox status in type 1 diabetic mice [73]. At the same time, we will face critical challenges in clinical translation due to drug toxicity and organ-specific targeting. Currently, simultaneous delivery to renal glomeruli and cardiac myocardium remains technically challenging. Site-specific nanoparticles or antibody conjugates may be necessary [74, 75]. These drugs are currently mainly used for other indications, such as cancer, and their potential role in modulating key signaling pathways in DKD and DCM warrants further investigation. Future studies should focus on validating these drug-gene interactions in clinical settings and exploring their therapeutic efficacy.

While our study provides significant insights into the shared molecular mechanisms of DKD and DCM, several limitations need to be acknowledged. First, the GEO datasets employed have relatively small sample sizes (GSE30122: 9 DKD *vs*. 26 controls; GSE197850: 4 DCM *vs*. 4 controls) and inherent heterogeneity across different experimental platforms. The relatively small sample sizes, particularly within the DCM dataset (GSE197850), may limit the statistical power and generalizability of the identified DEGs. Expanding the analysis to include additional datasets and using standardized platforms could improve the robustness of the findings. Second, this study confirmed the dysregulation of hub gene expression at the mRNA level using *in vitro* (AGEs-stimulated H9C2 cardiomyocytes and MPC5 podocytes) and *in vivo* (STZ-induced rat models of DCM and DKD) models. However, functional validation of the identified hub genes through techniques, such as knockdown (*e.g.*, siRNA/shRNA) or overexpression in relevant cell models, is lacking. Such experiments are crucial to definitively establish the causal roles of these hub genes in the pathogenesis of the DCM and DKD phenotypes observed *in vitro* (*e.g.*, changes in cell viability) and *in vivo* (*e.g.*, histological damage, functional impairment). Third, our *in vivo* models employed STZ-induced diabetes. While widely accepted, STZ models primarily reflect the pathophysiology of type 1 diabetes. Further investigation into models representing type 2 diabetes and its comorbidities would strengthen the translational relevance of our findings.

## CONCLUSION

Through integrated bioinformatics analysis and experimental verification, this study identified seven common core genes (CD200, CRHBP, DHRS3, EMCN, HPGD, PDGFRB, and SULF1) in diabetes cardiomyopathy (DCM) and diabetes nephropathy (DKD). Both *in vitro* (AGE-stimulated cardiomyocytes and podocytes) and *in vivo* (diabetic rat model) experiments confirmed that these genes were significantly downregulated in both complications. Based on these core genes, we screened 10 potential therapeutic compounds (*p*-adj < 0.05) using computational drug repositioning analysis. These findings suggest that the seven hub genes may jointly participate in the pathophysiological processes of DCM and DKD by regulating pathways, such as angiogenesis, extracellular matrix remodeling, and the inflammatory response.

## Figures and Tables

**Fig. (1):**
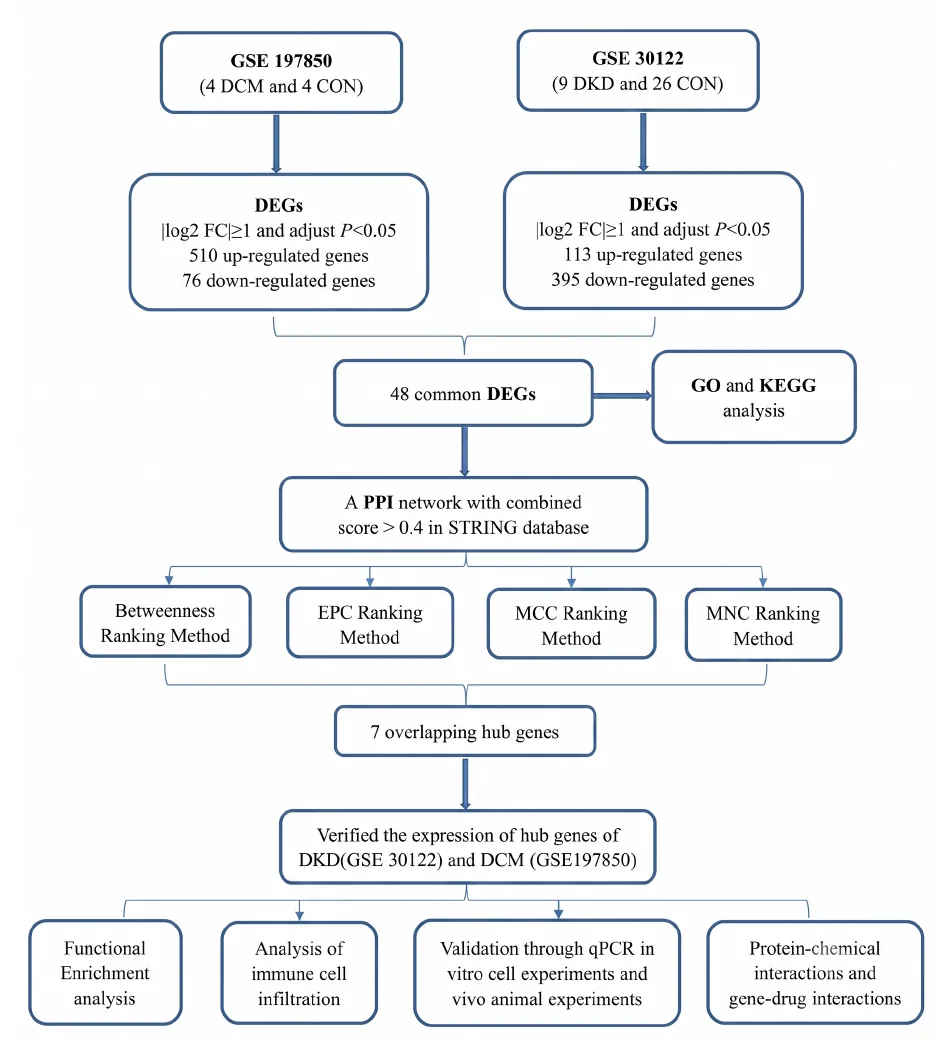
Flowchart depicting cross-disease analysis of diabetic cardiomyopathy (DCM, GSE197850: 4 cases *vs*. 4 controls) and diabetic kidney disease (DKD, GSE30122: 9 cases *vs*. 26 controls) datasets. Key steps: DEG screening (|log_2_FC|≥1, adjusted *p*<0.05) identifying 510↑/76↓ (DCM) and 113↑/395↓ (DKD) genes; Venn analysis revealing 48 shared DEGs; functional annotation (GO/KEGG) and STRING-based PPI network (score>0.4); hub gene identification using four topological methods (Betweenness/EPC/MCC/MNC); validation through cross-dataset verification, qPCR experiments, immune infiltration analysis, and therapeutic target mapping.

**Fig. (2):**
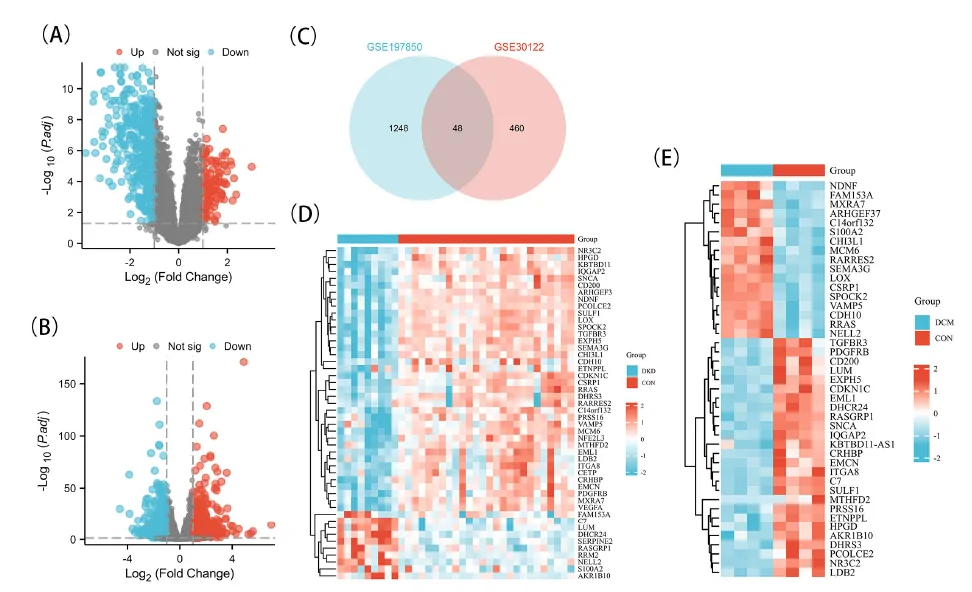
Differentially expressed genes (DEGs) in DKD and DCM datasets. (**A**) Volcano plot of DEGs in the GSE 30122 dataset. Red color indicates up-regulated genes and blue color indicates down-regulated genes. (**B**) Volcano plot of DEGs in the GSE 197850 dataset. Red color indicates up-regulated genes and blue color indicates down-regulated genes. (**C**) Venn diagram of overlapping DEGs among two GEO datasets. (**D**) Heat maps of overlapping DEGs in the GSE 30122 dataset. (**E**) Heat maps of overlapping DEGs in the GSE 197850 dataset.

**Fig. (3):**
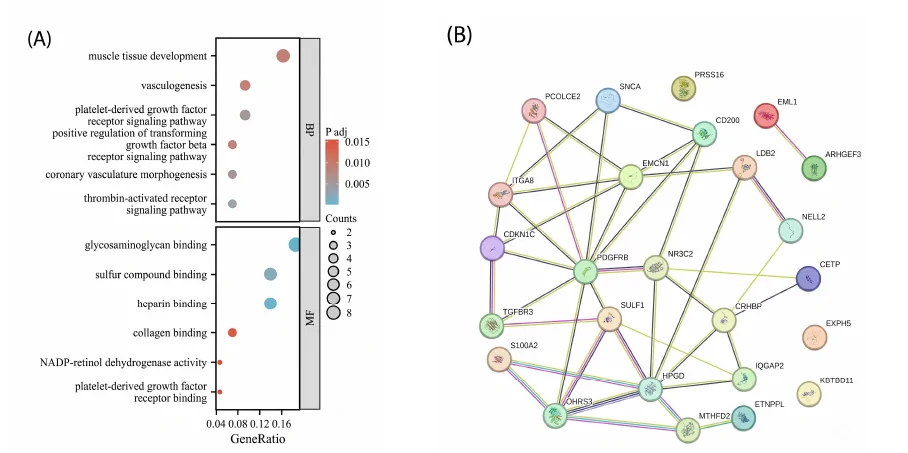
Functional enrichment analysis and protein-protein interaction. (**A**) Gene ontology (GO) enrichment analysis of 48 overlapping DEGs. (**B**) Protein-protein interaction of the overlapping DEGs. Nodes represent proteins and edges denote predicted interactions, with edge thickness reflecting interaction confidence.

**Fig. (4):**
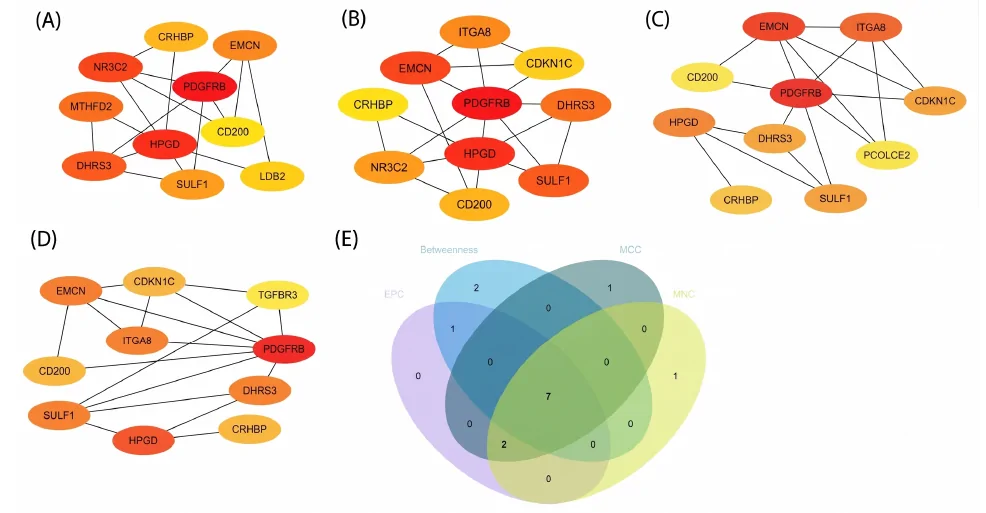
Identification of Hub genes in overlapping DEGs among two GEO datasets. (**A**) The betweenness ranking method for hub genes identification. (**B**) The edge percolated component (EPC) ranking method for hub gene identification. (**C**) The maximum clique centrality (MCC) ranking method for hub gene identification. (**D**) The maximum neighborhood component ranking (MNC) method for hub gene identification. (**E**) Venn diagram for identifying hub genes among different ranking methods.

**Fig. (5):**
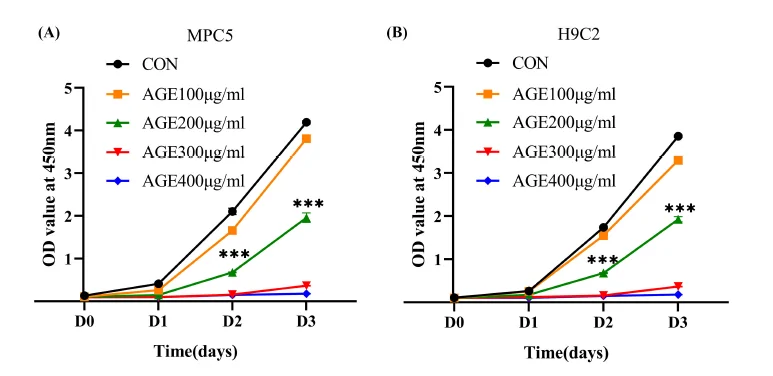
The results of the Cell Counting Kit (CCK)-8 assay. (**A**) MPC5 podocytes and (**B**) H9C2 cardiomyocytes exposed to 0-400 μg/mL AGEs for 72 hours (D0-D3) show time- and concentration-dependent OD450 elevation. Three independent experiments (n=3) demonstrated significant proliferative effects starting at 200 μg/mL (***p<0.001 *vs*. CON, two-way ANOVA with Tukey's test).

**Fig. (6):**
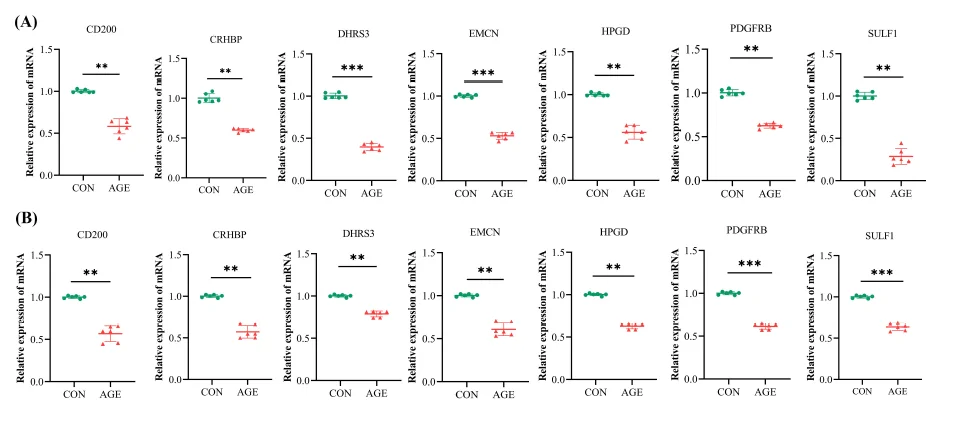
The results of qRT-PCR in the group of AGE-treated MPC5 and H9C2. Data are presented as mean± SEM. Statistical significance was determined by an unpaired Student’s t-test: **p<0.01, ***p<0.001 *versus* control. (**A**) Relative expression of mRNA between the control and AGEs-treated MPC5. (**B**) Relative expression of mRNA between the control and AGEs-treated H9C2.

**Fig. (7):**
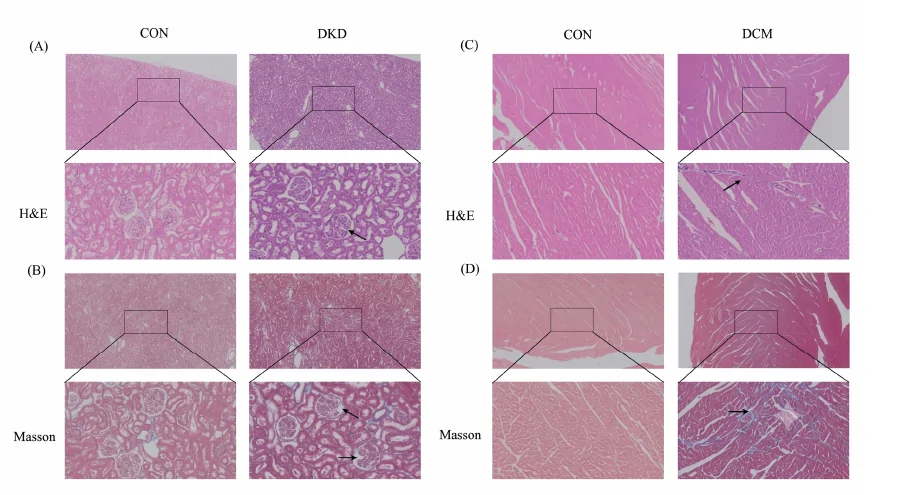
Hematoxylin and eosin (HE) staining and Masson staining images of rat kidneys and hearts in each group. The characteristic lesion has been marked with black arrows. (**A**) HE-stained kidney section from the control group and the DKD group. (**B**) Masson-stained kidney section from the control group and the DKD group. (**C**) HE-stained myocardial section from the control group and the DCM group. (**D**) Masson-stained myocardial section from the control group and the DCM group. The entire section is presented at 5× magnification, and the magnified area is at 20× magnification.

**Fig. (8):**
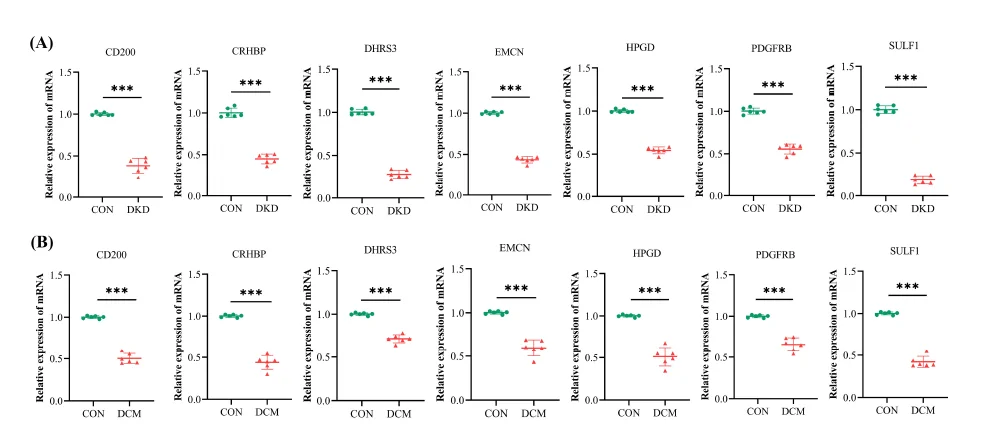
The results of qRT-PCR in the group of DKD rats and DCM rats. Data are presented as mean ± SEM. Statistical significance was determined by an unpaired Student’s t-test: ***p<0.001 *versus* control. (**A**) Relative expression of mRNA between the control group and the DKD group. (**B**) Relative expression of mRNA between the control group and the DCM group.

**Fig. (9):**
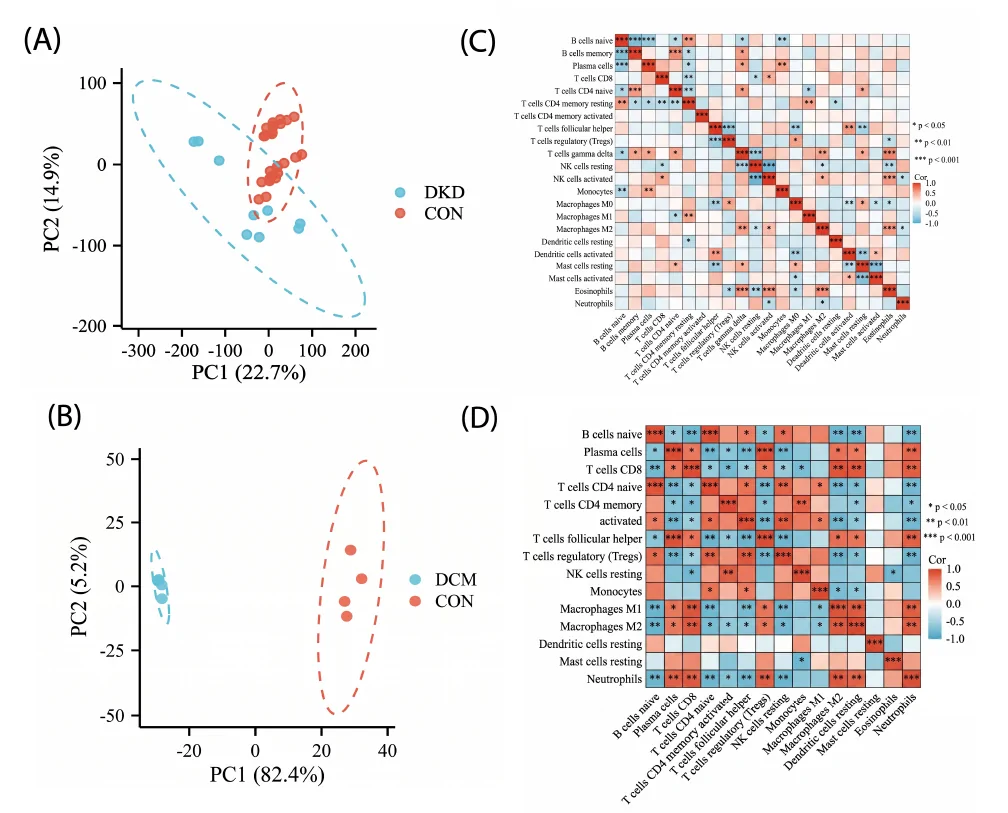
Visualization of immune cell infiltration in both the DKD group and the DCM group. (**A**) PCA plot of immune cell infiltration between the DKD group and the control group samples. (**B**) PCA plot of immune cell infiltration between the DCM group and the control group samples. (**C**) Correlation heat map between different kinds of immune cells in DKD group samples. (**D**) Correlation heat map between different kinds of immune cells in DCM group samples. The abscissa and ordinate represent different immune cells; different colors represent different correlation coefficients (Red: positive correlation; blue: negative correlation). The darker the color, the stronger the relationship.

**Fig. (10):**
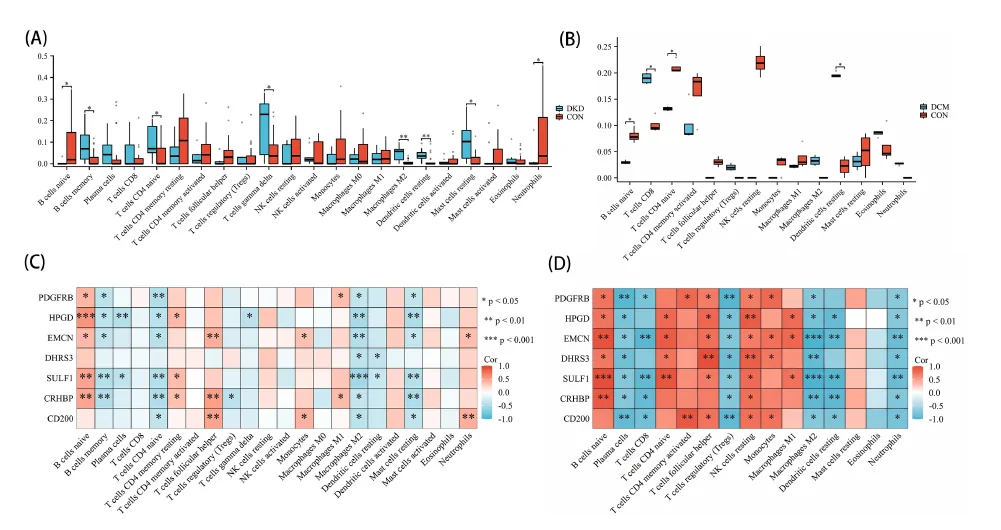
DKD and DCM are associated with immune infiltration. (**A**) The difference in immune infiltration proportions between the DKD group and the control group samples. (**B**) The difference in immune infiltration proportions between the DCM group and the control group samples. *p < 0.05, **p < 0.01, ***p < 0.001; asterisks (*) stand for significance levels. The statistical difference between the two groups is compared through the Wilcoxon rank-sum test. (**C**) The correlation between immune cell infiltration and the expression of CD200, CRHBP, DHRS3, EMCN, HPGD, PDGFRB, and SULF1 in the DKD cohort. (**D**) The correlation between immune cell infiltration and the expression of CD200, CRHBP, DHRS3, EMCN, HPGD, PDGFRB, and SULF1 in the DCM cohort. Red and blue colors indicate positive and negative correlations, respectively; darker shades represent stronger correlations.

**Fig. (11):**
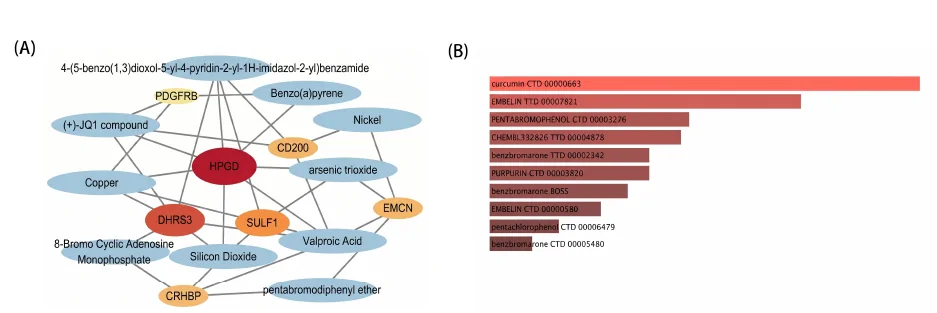
Protein-chemical interaction and gene-drug interaction. (**A**) Protein-chemical interaction network (red indicates proteins, while blue indicates chemicals). (**B**) Bar chart of the top 10 drugs that are associated with seven hub genes.

**Table 1:** The details of the intersected hub genes.

**Gene Symbol**	**Full Name**	**Description**
CD200	CD200 molecule	This gene encodes a type I membrane glycoprotein containing two extracellular immunoglobulin domains, a transmembrane and a cytoplasmic domain. This gene is expressed by various cell types, including B cells, a subset of T cells, thymocytes, endothelial cells, and neurons. The encoded protein plays an important role in immunosuppression and regulation of anti-tumor activity.
CRHBP	Corticotropin releasing hormone binding protein	Corticotropin-releasing hormone is a potent stimulator of synthesis and secretion of preopiomelanocortin-derived peptides. Although CRH concentrations in the human peripheral circulation are normally low, they increase throughout pregnancy and fall rapidly after parturition. Maternal plasma CRH probably originates from the placenta. Human plasma contains a CRH-binding protein which inactivates CRH and which may prevent inappropriate pituitary-adrenal stimulation in pregnancy.
DHRS3	Dehydrogenase/reductase 3	Predicted to enable NAD-retinol dehydrogenase activity. Predicted to be involved in regulation of retinoic acid receptor signaling pathway and retinoid metabolic process. Predicted to act upstream of or within several processes, including animal organ morphogenesis; negative regulation of retinoic acid receptor signaling pathway; and regulation of ossification. Predicted to be located in endoplasmic reticulum membrane and photoreceptor outer segment membrane. Predicted to be active in lipid droplet.
EMCN	Endomucin	EMCN is a mucin-like sialoglycoprotein that interferes with the assembly of focal adhesion complexes and inhibits interaction between cells and the extracellular matrix.
HPGD	15-Hydroxyprostaglandin dehydrogenase	This gene encodes a member of the short-chain nonmetalloenzyme alcohol dehydrogenase protein family. The encoded enzyme is responsible for the metabolism of prostaglandins, which function in a variety of physiologic and cellular processes such as inflammation. Mutations in this gene result in primary autosomal recessive hypertrophic osteoarthropathy and cranioosteoarthropathy. Multiple transcript variants encoding different isoforms have been found for this gene.
PDGFRB	Platelet derived growth factor receptor beta	The protein encoded by this gene is a cell surface tyrosine kinase receptor for members of the platelet-derived growth factor family. These growth factors are mitogens for cells of mesenchymal origin. This gene is essential for normal development of the cardiovascular system and aids in rearrangement of the actin cytoskeleton. This gene is flanked on chromosome 5 by the genes for granulocyte-macrophage colony-stimulating factor and macrophage-colony stimulating factor receptor.
SULF1	Sulfatase 1	This gene encodes an extracellular heparan sulfate endosulfatase. The encoded enzyme selectively removes 6-O- sulfate groups from heparan sulfate chains of heparan sulfate proteoglycans (HSPGs). The enzyme is secreted through the Golgi and is subsequently localized to the cell surface. The expression of this gene may be down-regulated in several types of cancer, including hepatocellular (HCC), ovarian and breast cancers.

**Table 2:** Suggested top 10 chemicals for hub genes.

**Id**	**Compound**		**Degree**		**Betweenness**		**Chemical Formula**		**Structure**	
D014635	Valproic Acid		6		26.83		C_8_H_16_O_2_		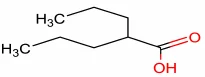	
C561695	(+)-JQ1 compound		4		10.39		C_23_H_25_ClN_4_O_2_S		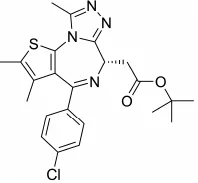	
D012822	Silicon Dioxide		4		7.98		SiO_2_		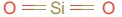	
D003300	Copper		4		7.89		Cu		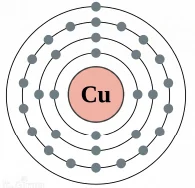	
C459179	4-(5-benzo(1,3)dioxol-5-yl-4-pyridin-2-yl-1H-imidazol-2-yl)benzamide		4		5.99		C_7_H_7_NO		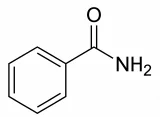	
C006632	Arsenic trioxide		3		5.63		As_2_O_3_		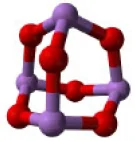	
C086401	Pentabromodiphenyl ether		2		1.74		C_12_H_5_Br_5_O		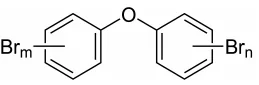	
D009532	Nickel		2		1.60		Ni		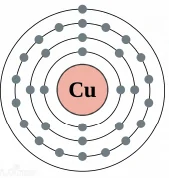	
D001564	Benzo(a)pyrene		2		1.48		C_20_H_12_		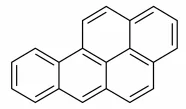	
D015124	8-Bromo Cyclic Adenosine Monophosphate		2		1.47		C_10_H_11_BrN_5_O_6_P		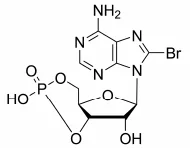	

**Note:** Based on the degree of connectivity of protein-chemical interactions, the ID, name, equation and structural formula of the top 10 chemicals are presented.

**Table 3:** Suggested top 10 drugs for DN combined DCM.

**Drug Name**	***p*-value**	**Chemical Formula**	**Structure**	**Genes**
Curcumin CTD 00000663	1.58E-05	C_21_H_20_O_6_	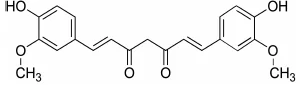	PDGFRB;CRHBP;HPGD;SULF1
Embelin TTD 00007821	3.40E-05	C_17_H_26_O_4_	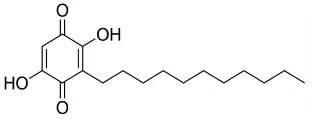	CRHBP;HPGD
Pentabromophenol CTD 00003276	6.95E-05	C_6_Br_5_OH	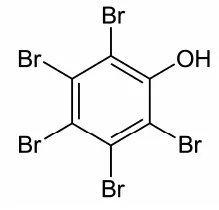	CRHBP;HPGD
Chembl 332826 TTD 00004878	7.34E-05	C_25_H_19_Cl_2_N_3_O_2_	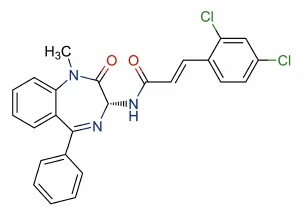	CRHBP;HPGD
Benzbromarone TTD 00002342	8.98E-05	C_17_H_12_Br_2_O_3_	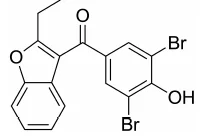	CRHBP;HPGD
Purpurin CTD 00003820	8.98E-05	C_14_H_8_O_5_	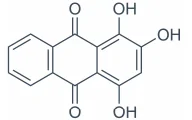	CRHBP;HPGD
Benzbromarone BOSS	1.03E-04	C_17_H_12_Br_2_O_3_	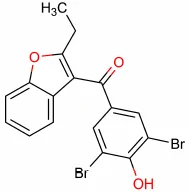	CRHBP;HPGD
Embelin CTD 00000580	1.23E-04	C_10_H_10_BrN_5_NaO_6_P	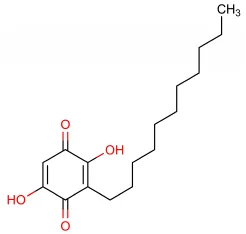	CRHBP;HPGD
Pentachlorophenol CTD 00006479	1.60E-04	C_6_HCl_5_O	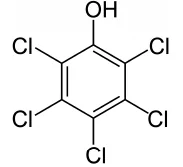	CRHBP;HPGD
Benzbromarone CTD 00005480	1.90E-04	C_17_H_12_Br_2_O_3_	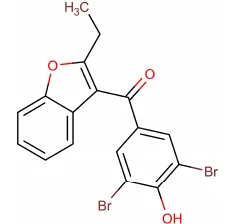	CRHBP;HPGD

**Note:** Based on the name, *p*-value, equation and structural formula of the top 10 drugs are presented.

## Data Availability

The datasets analyzed in our study were downloaded from the GEO database, and are available from the corresponding author upon reasonable request.

## LIST OF ABBREVIATIONS

DM: Diabetes Mellitus
DKD: Diabetic Kidney Disease
DCM: Diabetic Cardiomyopathy
GEO: Gene Expression Omnibus
DEGs: differentially Expressed Genes
qPCR: Quantitative Real-time PcR
ESRD: End-stage Renal Disease
KEGG: Kyoto Encyclopedia of Genes and Genomes
GO: Gene Ontology
BP: Biological Process
CC: Cell Component
MF: Molecular Function.
