# Supplementary material for: Decoding Diabetes: Hub Genes as Pivotal Players in Cardiomyopathy and Kidney Disease
Source: Endocr Metab Immune Disord Drug Targets. 2026 Jan 15;26:E18715303393363. doi: 10.2174/0118715303393363251028113435 (PMC13662719; doi:10.2174/0118715303393363251028113435)
Supplement: Supplementary file 1 [file EMIDDT-26-E18715303393363_SD1.pdf]

Supplementary Material

Decoding Diabetes: Hub Genes as Pivotal Players in Cardiomyopathy and Kidney Disease

Jiarong Liu[1,#], Wen Chen[1,#], Yun Zou[1] and Jixiong Xu[1,2,3,*]

[1]Department of Endocrine and Metabolism, The First Affiliated Hospital, Jiangxi Medical College, Nanchang University, Nanchang, Jiangxi, People's Republic of China; [2]Jiangxi Clinical Research Center for Endocrine and Metabolic Disease, Nanchang, Jiangxi, People's Republic of China; [3]Jiangxi Branch of National Clinical Research Center for Metabolic Disease, Nanchang, Jiangxi, People's Republic of China

Supplementary Table S1: Primer sequence of quantitative real-time PCR.

Species	Primer name	Primer sequence
Mouse	PDGFRB (Forward)	5'-AGGAGTGATACCAGCTTTAGTCC-3'
Mouse	PDGFRB (Reverse)	5'-CCGAGCAGGTCAGAACAAAGG-3'
Mouse	HPGD (Forward)	5'-CAGGGCATAGGCAAAGCCTT-3'
Mouse	HPGD (Reverse)	5'-ACGCCTGCATTGTTGACCAA-3'
Mouse	EMCN (Forward)	5'-AATACCAGGCATCGTGTCAGT-3'
Mouse	EMCN (Reverse)	5'-CCACTTCATGTTTTGGTGTTGTC-3'
Mouse	DHRS3 (Forward)	5'-TGAAAAAATGCCTCAAGGAGACG-3'
Mouse	DHRS3 (Reverse)	5'-GGCCATCTGGTACACCTCTTC-3'
Mouse	SULF1 (Forward)	5'-GTGCTCACTGACGACCAGG-3'
Mouse	SULF1 (Reverse)	5'-TGTAGACATTGTGGTTATGCACG-3'
Mouse	CRHBP (Forward)	5'-CTTACCCTCGGACACTTGCAT-3'
Mouse	CRHBP (Reverse)	5'-GGTCTGCTAAGGGCATCATCT-3'
Mouse	CD200 (Forward)	5'-TCTCCACCTACAGCCTGATTT-3'
Mouse	CD200 (Reverse)	5'-AGAACATCGTAAGGATGCAGTTG-3'
Mouse	GAPDH (Forward)	5'-AGGTCGGTGTGAACGGATTTG-3'
Mouse	GAPDH (Reverse)	5'-GGGGTCGTTGATGGCAACA-3'
Rat	PDGFRB (Forward)	5'-GCAACGACACTCCAACAA-3'
Rat	PDGFRB (Reverse)	5'-TCCCCAGTCAGGTTCAAG-3'
Rat	HPGD (Forward)	5'-GTAGCATTGGTGGATTGGA-3'
Rat	HPGD (Reverse)	5'-GGCCGCTTTACATTTTACAC-3'

Rat	EMCN (Forward)	5'-GGGAAAGCCAAGAACTGAC-3'
Rat	EMCN (Reverse)	5'-TTGCCCAGGTGTAAGGAG-3'
Rat	DHRS3 (Forward)	5'-CTGCACATCAAAAGCATCA-3'
Rat	DHRS3 (Reverse)	5'-TGCCCTGAAACATCTCG-3'
Rat	SULF1 (Forward)	5'-TTCACCAACGTCCTCCA-3'
Rat	SULF1 (Reverse)	5'-CCCTTGACCAGTCCAAAC-3'
Rat	CRHBP (Forward)	5'-CACCTCCAAGATGATGCTC-3'
Rat	CRHBP (Reverse)	5'-TCAAAAGTCACACGGTTCA-3'
Rat	CD200 (Forward)	5'-TCAAAGACCCCAAAACTCA-3'
Rat	CD200 (Reverse)	5'-GTCAATCACATTCCCCAAG-3'
Rat	GAPDH (Forward)	5'-ATGGCTACAGCAACAGGGT-3'
Rat	GAPDH (Reverse)	5'-TTATGGGGTCTGGGATGG-3'

Supplementary Table S2. Basic information for common DEGs.

Gene Symbol	log2FC(GSE197850)	log2FC(GSE30122)
AKR1B10	-1.4337344	1.733356942
ARHGEF3	-1.0479285	-1.826434223
C14orf132	1.1331321	-1.773978949
C7	-1.7273623	1.875416012
CD200	-1.2763821	-1.46396836
CDH10	2.0151829	-1.560197927
CDKN1C	-1.2435466	-2.503973568
CETP	-1.215289	-1.236954872
CHI3L1	1.5066973	-2.814794446
CRHBP	-1.9527025	-2.074767544
CSRP1	1.2985363	-1.584702693
DHCR24	-1.3436191	1.117657136
DHRS3	-1.4193217	-1.151995793
EMCN	-3.032504	-2.526809034

EML1	-1.034293	-1.327314984
ETNPPL	-1.8404361	-1.109795816
EXPH5	-1.1465764	-1.37992942
FAM153A	2.38428	-1.470598395
HPGD	-1.5035387	-1.477824122
IQGAP2	-2.3958792	-1.482211565
ITGA8	-1.2714591	-1.855607659
KBTBD11	-1.0192676	-1.57830332
LDB2	-1.8362427	-1.619343298
LOX	1.6209934	-2.224573193
LUM	-1.6583708	2.275878782
MCM6	1.1299081	-1.900771712
MTHFD2	-1.3117978	-1.506973885
MXRA7	1.0383161	-1.17539066
NDNF	1.0715569	-3.463522584
NELL2	1.0604607	1.021409465
NFE2L3	1.0131815	-1.486688174
NR3C2	-1.1999324	-1.063676209
PCOLCE2	-1.1117233	-3.114312001
PDGFRB	-1.3509403	-1.066402739
PRSS16	-1.3318533	-1.006388954
RARRES2	2.9837106	-1.003276786
RASGRP1	-1.3535942	1.346092241
RRAS	1.2683638	-1.902760764
RRM2	-1.1191304	1.295523268
S100A2	2.1039605	1.047184634
SEMA3G	2.8041207	-2.853964235
SERPINE2	-1.0134702	1.814586452
SNCA	-1.6077289	-1.674000607
SPOCK2	1.7217232	-1.800392487

SULF1	-1.4226967	-2.223814004
TGFBR3	-1.5818753	-2.356263428
VAMP5	1.8581721	-1.088254937
VEGFA	1.0706266	-1.808270816

Supplementary Table S3. GO analysis of common DEGs

Gene Ontology	Name	Count	*P* value	Genes
**Biological Processes**				
GO:0070493	Thrombin-activated receptor signaling pathway	3	2.41701e-06	SNCA/IQGAP2/HPGD
GO:0048008	Platelet-derived growth factor receptor signaling pathway	4	7.43225e-06	SNCA/LOX/PDGFRB/VEGFA
GO:0060977	Coronary vasculature morphogenesis	3	1.05276e-05	TGFBR3/PDGFRB/VEGFA
**Cellular Component**				
GO:0062023	Collagen-containing extracellular matrix	6	0.000474749	MXRA7/SULF1/LOX/RARRES2/LUM/SERPINE2
GO:0031091	Platelet alpha granule	3	0.001273128	SNCA/SERPINE2/VEGFA
**Molecular Functions**				
GO:0005539	Glycosaminoglycan binding	8	5.97356e-08	SPOCK2/SULF1/NDNF/TGFBR3/NELL2/PCOLCE2/SERPINE2/VEGFA
GO:0008201	Heparin binding	6	2.43394e-06	NDNF/TGFBR3/NELL2/PCOLCE2/SERPINE2/VEGFA
GO:1901681	Sulfur compound binding	6	3.41864e-05	NDNF/TGFBR3/NELL2/PCOLCE2/SERPINE2/VEGFA

Notes: Functional and pathway enrichment analyses for module genes. The top 3 terms were selected based upon *p* value rankings when >3 terms were enriched for a given category.

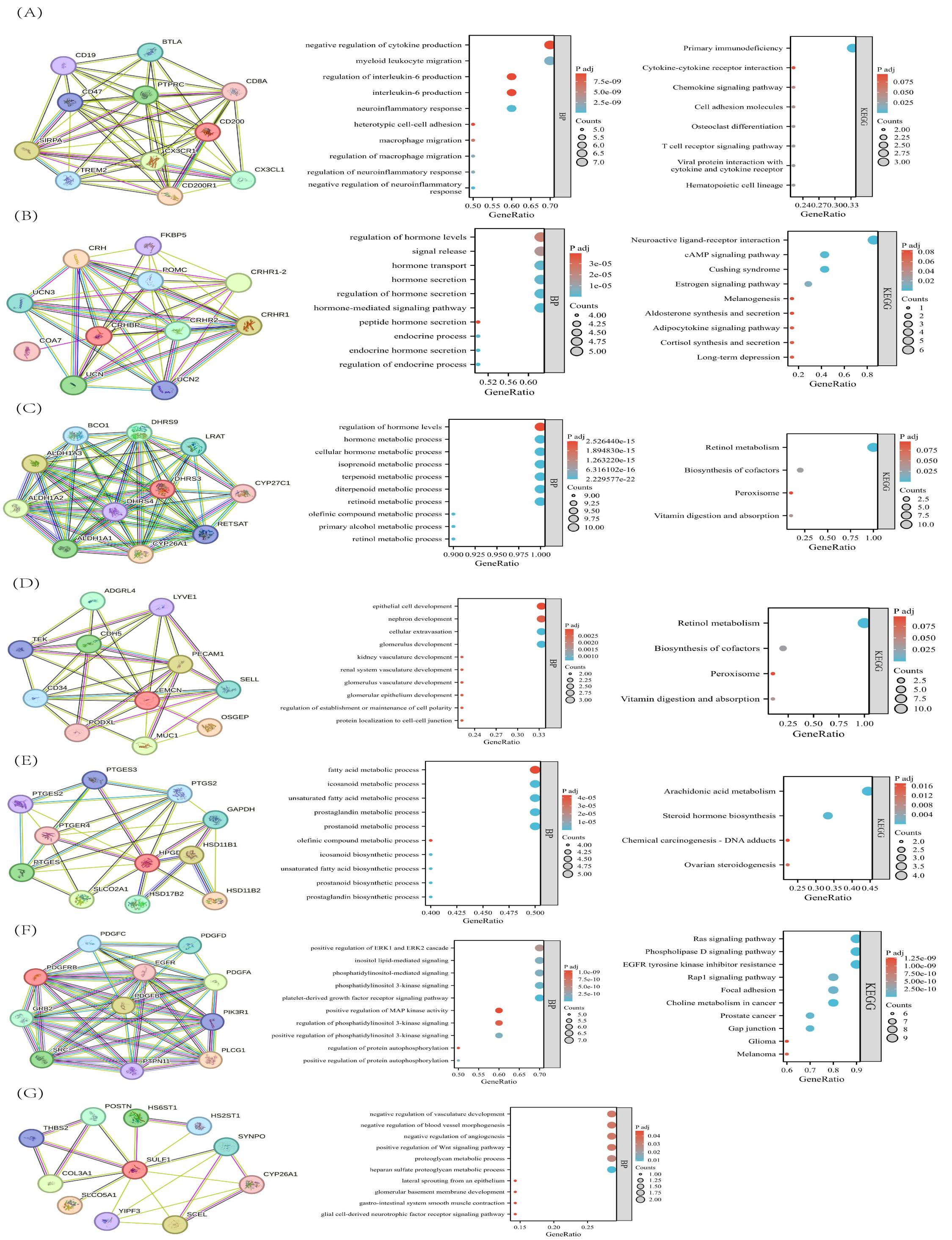

Supplementary Figure 1: Functional enrichment analysis of proteins interacting with 7 hub genes. (**A**) Protein interaction network of CD200. BP and KEGG enrichment analysis of the protein interaction network of CD200. (**B**) Protein interaction network of CRHBP. BP and KEGG enrichment analysis of the protein interaction network of CRHBP. (**C**) Protein interaction network of DHRS3. BP enrichment analysis of the protein interaction network of DHRS3. (**D**) Protein interaction network of EMCN. BP and KEGG enrichment analysis of the protein interaction network of EMCN. (**E**) Protein interaction network of HPGD. BP and KEGG enrichment analysis of the protein interaction network of HPGD. (**F**) Protein interaction network of PDGFB. BP and KEGG enrichment analysis of the protein interaction network of PDGFB. (**G**) Protein interaction network of SULF1. BP and KEGG enrichment analysis of the protein interaction network of SULF1.
